# Using Crowdsourcing Technology for Testing Multilingual Public Health Promotion Materials

**DOI:** 10.2196/jmir.2063

**Published:** 2012-06-04

**Authors:** Anne M Turner, Katrin Kirchhoff, Daniel Capurro

**Affiliations:** ^1^Department of Health ServicesUniversity of WashingtonSeattle, WAUnited States; ^2^Department of Biomedical Informatics and Medical EducationUniversity of WashingtonSeattle, WAUnited States; ^3^Department of Electrical EngineeringUniversity of WashingtonSeattle, WAUnited States; ^4^Department of Internal MedicinePontificia Universidad Catolica de ChileSantiagoChile

**Keywords:** Crowdsourcing, health promotion, public health informatics, limited English proficiency populations

## Abstract

**Background:**

Effective communication of public health messages is a key strategy for health promotion by public health agencies. Creating effective health promotion materials requires careful message design and feedback from representatives of target populations. This is particularly true when the target audiences are hard to reach as limited English proficiency groups. Traditional methods of soliciting feedback—such as focus groups and convenience sample interviews—are expensive and time consuming. As a result, adequate feedback from target populations is often insufficient due to the time and resource constraints characteristic to public health.

**Objective:**

To describe a pilot study investigating the use of crowdsourcing technology as a method to gather rapid and relevant feedback on the design of health promotion messages for oral health. Our goal was to better describe the demographics of participants responding to a crowdsourcing survey and to test whether crowdsourcing could be used to gather feedback from English-speaking and Spanish-speaking participants in a short period of time and at relatively low costs.

**Methods:**

We developed health promotion materials on pediatric dental health issues in four different formats and in two languages (English and Spanish). We then designed an online survey to elicit feedback on format preferences and made it available in both languages via the Amazon Mechanical Turk crowdsourcing platform.

**Results:**

We surveyed 236 native English-speaking and 163 native Spanish-speaking participants in less than 12 days, at a cost of US $374. Overall, Spanish-speaking participants originated from a wider distribution of countries than the overall Latino population in the United States. Most participants were in the 18- to 29-year age range and had some college or graduate education. Participants provided valuable input for the health promotion material design.

**Conclusions:**

Our results indicate that crowdsourcing can be an effective method for recruiting and gaining feedback from English-speaking and Spanish-speaking people. Compared with traditional methods, crowdsourcing has the potential to reach more diverse populations than convenience sampling, while substantially reducing the time and cost of gathering participant feedback. More widespread adoption of this method could streamline the development of effective health promotion materials in multiple languages.

## Introduction

Effective communication of health messages to a wide range of populations is a key public health strategy for preventing disease. Unfortunately, the vast majority of good-quality health information materials—such as websites, flyers, and patient handouts—in the United States are available only in English. Communication efforts frequently do not reach diverse populations because of language barriers. Design of effective health promotion materials for linguistically diverse populations requires careful development of key messages, as well as evaluation and feedback from target communities. Most methods that require recruitment of participants to develop and test multilingual health messages are both costly and time consuming [1]. We report here on the use of crowdsourcing to gather quick feedback on health promotion materials from English-speaking and Spanish-speaking populations in an inexpensive and efficient manner. The specific advantages and challenges of using this technique for health communications research are explored.

### Background and Motivation

Health promotion materials—either in print or online—are important vehicles for communicating public health messages. Prior studies indicated that the design of these materials significantly affects readers’ understanding and retention of these messages [2,3]. In practice, however, too little attention is paid to information selection, wording, formatting, and the use of different modalities (text vs images). The effectiveness of different materials may also depend on the native language and cultural background of the target audience [4,5]. This factor should not be underestimated: according to the American Community Survey [6], 20% of the US population over 5 years of age speak a language other than English at home, and 43.8% of these have limited English proficiency, defined as having a primary language other than English and a limited ability to read, speak, write, or understand English.

Two reasons why health promotion materials are often developed in a cursory manner are time and costs. Typical health promotion research is conducted through surveys, interviews, and focus groups [7,8]. Such studies are costly, time consuming, often subject to selection bias, and of limited generalizability [9], since they most frequently rely on convenience samples of target populations.

Over the last 10 years, the use of Internet surveys has risen in popularity as a result of the ease of access and low costs. Although Internet surveys have raised concerns about generalizability, published studies indicate that Internet surveys that depend on self-selected populations reach more diverse populations than do traditional survey methods that rely on convenience samples. An investigation of a large sample of Internet participants (n = 361,703) revealed greater participant diversity in terms of gender, race, age, geographic diversity, and economic status than in traditional studies [10]. Crowdsourcing platforms provide a potential channel for easy access to and recruitment of participants for conducting Internet surveys, especially when trying to access a more diverse population such as non-English speakers. In this paper we communicate our experience using crowdsourcing technology to test public health promotion materials.

### Crowdsourcing

Crowdsourcing is a term used to describe the outsourcing of online tasks performed by a network of people responding to an open call [11]. Although crowdsourcing was initially used for assigning computer-coding tasks, its application has been expanded to product development, advertising, and marketing research [12]. Crowdsourcing, as a method to reach participants, is beginning to revolutionize fields that rely on human experts to perform complex tasks. Collecting data from participant experts has traditionally been difficult, slow, and costly. Examples include speech transcription [13], translations [14], and image labeling [15]. Of the existing online crowdsourcing platforms, the most well known and well studied is Amazon’s Mechanical Turk (www.Mturk.com). Mechanical Turk is a crowdsourcing website for brokering so-called human intelligence tasks (HITs)—that is, tasks that are easy for a human to perform but are difficult for a computer. Mechanical Turk connects requesters of HITs to workers and allows for easy task creation, recruitment, compensation, and data collection. The site provides 24/7 access to participants from over 100 countries.

### Crowdsourcing in Social Science Research

Since crowdsourcing provides easy, low-cost access to a potentially large pool of participants, it is starting to be considered as a method for research study recruitment in the psychological and behavioral sciences. Mechanical Turk and similar platforms can be used to conduct surveys, opinion polls, or online experiments. Crowdsourcing differs from traditional Internet surveys in that involves limited financial compensation and a pool of regular participants; thus, the recruitment potential, in terms of speed and number of participants, is greatly increased. The potential concern with this method is that there is no face-to-face interaction with participants—the natural question that arises is whether experimental results obtained in this way are valid. Participants might try to “game” the system and not be sufficiently engaged in the task, especially if their primary motivation is monetary compensation. In view of these concerns (see Schmidt [16] for a discussion), several recent studies have analyzed the validity of Mechanical Turk-based data collection for human participants research in political science, psychology, economics, and linguistics [17-21]. These studies have unanimously found that data gathered through Mechanical Turk closely mirrored results collected using standard experimental paradigms, demonstrating a high level of participant engagement, while being significantly easier, faster, and cheaper to obtain.

 The relatively low level of compensation typical of Mechanical Turk HITs affected data collection time but not data quality. When compared with convenience samples and other Internet surveys, Mechanical Turk provided data that appeared to be as reliable as other traditional methods [17]. One reason for this outcome may be that, at least for workers located within the United States, the main motivations for working on Mechanical Turk are spending free time in a useful way, having fun, and earning additional income [22,23]. This is different for workers in other countries (eg, India, which hosts the second-largest segment of Mechanical Turk workers) where compensation is a stronger motivation, though few people use Mechanical Turk as their primary source of income [17]. Berinsky et al [18] and Buhrmester et al [19] also found that Mechanical Turk participants are more diverse and demographically representative than convenience samples.

### Crowdsourcing in Public Health

In the context of health care, various forms of crowdsourcing have been used for disaster response [24] and reporting disease outbreaks [25]. These forms of crowdsourcing relied on unpaid volunteers to provide services such as language translation or geolocation. To the best of our knowledge, Mechanical Turk or comparable platforms that implement online microtask brokering have not been studied as a tool in public health communications research or practice, in particular for testing and validating user-oriented health information materials.

Our hypothesis was that crowdsourcing could be an alternative to in-person methods to test public health promotion materials, especially to gain access to non-English-speaking populations. We set out to determine the potential of Mechanical Turk as a rapid, low-cost method for testing the format of health promotion messages designed for diverse populations, including limited English proficiency populations. In particular, we sought to identify the ease of recruitment, costs, and participant demographics associated with using Mechanical Turk to gather rapid and relevant feedback regarding formatting preference from English-speaking and Spanish-speaking individuals for public health communications research.

For this tutorial we chose to focus on our experience studying pediatric dental health messages. Despite being highly preventable, dental disease remains the most common disease of children and adolescents [26]. Tooth decay is four times more common than asthma among adolescents aged 14 to 17 years. Over the past 50 years, major improvements in dental health have been reported nationally in the United States, yet striking disparities remain based on income, age, and race or ethnicity [26]. Many members of society are not informed about, or do not act upon, available dental health messages; therefore, we viewed dental health messaging as an important area for investigating targeted communications.

## Methods

### Mechanical Turk Setup

Amazon Mechanical Turk facilitates several steps in a crowdsourcing-based study, in particular publishing the task, recruiting participants, collecting the data, and compensating workers. Data preparation and response quality assessment need to be done offline by the researcher.

The first step in setting up a crowdsourcing task is to create and fund a Mechanical Turk account. There is no cost associated with setting up an account, but funds to compensate the workers and to pay the nominal fees charged by the website need to be paid into the account in advance.

The next step involves setting up the task to be performed by workers. The study designer needs to define the overall task, break it up into microtasks (small tasks that can quickly be performed by an individual worker), formulate the instructions to workers, and prepare the data associated with each task (such as text or images to be annotated, or survey questions). This is done offline, using in-house tools. The Mechanical Turk infrastructure is then used to determine the design of the HIT webpage presented to workers, as well as task and worker attributes, and to upload the data to Mechanical Turk. The desired number and type of HITs are then created automatically from the uploaded data and design template, and they are offered to workers meeting the specified attributes. For example, a researcher might want to annotate 100 different paragraphs. In this case, the template is the form designed to display the paragraph and capture the worker’s annotation. Then the data—the 100 different paragraphs—are loaded to create 100 individual HITs.

Task design and attribute specification can be performed using one of three alternatives: the Web-based requester interface, command-line tools, or an application programming interface. In each case, several predefined options are available for the page design (including, for example, checkboxes, drop-down menus, radio buttons, and free-text answers). The task attributes include the compensation per task, number of days the task will be available on Mechanical Turk, the maximum time allotted to any individual worker for completing the task once he or she has accepted it, the number of assignments per task (how many different workers process a given task), and the autoapproval period (the time period after which the results submitted by the worker will automatically be approved). The worker attributes include his or her approval rating (based on previous HITs completed on Mechanical Turk), geographic location, adult content qualification, and any additional qualifications set up by the requester (such as performance on previous tasks by the same requester). The set of worker attributes allows requesters to cultivate pools of trusted workers who habitually deliver good-quality results.

As soon as the template data collection form is created and the data are loaded, researchers can publish the HITs and start receiving answers from workers. Responses can be downloaded, assessed for quality, and approved or rejected online or by uploading a corresponding data file. Once a HIT has been approved, the worker is paid the promised compensation; requesters also have the option of assigning bonuses to workers for particularly satisfying results.

### Validity of Responses

One of the main difficulties faced when conducting crowdsourcing studies is assuring the validity of the responses obtained [27]. Since participation is anonymous and linked to monetary incentives, crowdsourcing can attract participants who do not fully engage in the requested tasks or might be unqualified to accurately complete them. There are several ways that a researcher might address this validity issue. The first one is one we already mentioned: setting up qualifications, including qualification tests that need to be passed before the worker can accept a HIT. Second, when the task is associated with an objective ground-truth answer for a subset of the data (such as finding a particular image among a set of images), responses can be rejected automatically when they do not correspond to the ground truth, and the worker can be blocked. However, this is not possible when the worker’s task is to provide a purely subjective assessment. Third, crowd-sourced data collection can involve multiple sequential stages—at each stage, a different set of workers correct the output from previous workers. Fourth, different measures of reliability can be computed on the responses offline, such as outlier statistics or agreement between multiple workers performing the same tasks. Finally, sanity checks (eg, comprehension questions) can be included in the HIT itself.

### Crowdsourcing in the Development of Health Promotion Materials

We developed an online survey to test formatting and modality preferences for a variety of messages on pediatric dental health issues (see Multimedia Appendix 1).

The survey consisted of three sections. In the first part we asked a set of questions about the participants’ demographic background, including country of origin, native language, age range, gender, highest education level achieved, whether participants had a regular dentist, and when they last saw a dentist. In the second part, described in more detail below, a paragraph extracted from a pediatric dental education document was presented in four different formats along with text comprehension questions. In the third part participants were asked to select which of the four formats they preferred, followed by an open-text question asking them to state the reasons for their preferences. Optionally, participants were able to provide feedback on the task itself.

In total, we created 12 different survey forms for 12 different documents, each about a different dental health topic. Consent to participate, including information about time to complete the survey and information being collected, was provided prior to initiating the survey. We did not collect any personally identifiable information during the survey; workers are anonymous and only associated with an alphanumerical identification tag. The University of Washington Human Subjects Division approved the study.

For parts 2 and 3 we selected paragraphs from consumer education materials available on US national dental association websites, including the National Institute of Dental and Craniofacial Research, the American Dental Association, the American Academy of Pediatric Dentistry, and the American Academy of Family Physicians. We selected paragraphs to represent a variety of topics regarding childhood dental health, such as tooth brushing, pediatric dental visits, or fluoride use. The content of the selected paragraph was formatted into four versions. Format A consisted only of the running-text paragraph. Format B was a text-only bulleted list. Format C showed the running-text paragraph and a content-related image (either a photorealistic image or graphics). Format D showed the bulleted list plus the image. All four formats were displayed on the same page. However, the order in which the four formats were presented was determined by random selection. To ensure that participants read and reviewed each of the four versions thoroughly, thus ensuring the validity of their responses, they were requested to answer a different text comprehension question after the presentation of each format. If they answered questions incorrectly, their responses were discarded. We created and tested two versions of the survey, one in English and one in Spanish.

 For each survey form, we created a separate HIT on Mechanical Turk. For each HIT, we collected 20 responses (ie, up to 20 different workers answered a single HIT, but a single HIT could not be completed multiple times by the same individual). For each of the two surveys we thus obtained 240 responses.

Participation was limited to individuals located in the United States and those 18 years or older. For the Spanish survey, participants were required to be native Spanish speakers and were asked to specify their country of origin. A separate language qualification test was not applied; however, all Spanish survey materials, including the HIT description and the comprehension questions, were in Spanish, and we did not see any evidence of nonnative speakers taking the Spanish survey. In addition, all comprehension questions were answered correctly. To ensure reliable participants, we also required that they have an approval rate of at least 95% in the HITs they had previously worked on. We allocated 15 minutes for the completion of a single HIT, although we estimated that it could be completed in a much shorter time; the compensation was US $0.25 per HIT.

## Results

The data gathered allowed us to gain insights into participant demographics, the time and costs related to conducting a Mechanical Turk survey, and users’ preferences for different messaging formats.

### Participant Demographics

We received responses from 236 individual participants for the English survey and 163 for the Spanish survey. Although participation was limited to individuals located in the United States, native Spanish-speaking participants were from 18 different countries. This is a wider distribution over countries than the overall Latino population in the United States, which tends to come more predominantly from Mexico, Central America, and the Caribbean [28]. The five most frequently mentioned countries included three countries in South America (Table 1).

**Table 1:** Most frequent countries of origin for native Spanish-speaking participants versus the US Latino population.

Study participants (n = 163)		US Latino population	
Country of origin	%	Country of origin	%
Mexico	20.9%	Mexico	63%
Colombia	9.4%	Puerto Rico	9.2%
Argentina	9.2%	Cuba	3.5%
Peru	6.1%	El Salvador	3.3%
El Salvador	4.6%	Dominican Republic	2.8%

Table 2 summarizes key demographic characteristics and the time required to answer our HITs. The overall demographic composition of our respondent populations is similar to compositions observed in previous studies [22,29]—that is, Mechanical Turk workers were predominantly young and well educated. It is noteworthy that the Spanish-speaking respondents overall seem to have been even more highly educated than their English-speaking counterparts.

**Table 2:** Comparison of Spanish-speaking and English-speaking survey participants.

Demographic characteristic	Spanish-speaking (n = 163)	English-speaking (n = 236)	*P *value
Time to completion of HITs^a^	~12 days	~6 days	NA^b^
Females, n (%)	73 (45%)	138 (58.5%)	.007
Age 18–40 years, n (%)	130 (79.8%)	184 (78%)	.67
College or graduate degree, n (%)	117 (71.8%)	102 (43.2%)	<.001
Average time/HIT (minutes:seconds), mean (SD)	5:20 (2:50)	4:05 (3:31)	<.001

^a ^Human intelligence tasks.

^b ^Not applicable.

### Time and Cost

The responses for the English survey were collected within 6 days; the Spanish survey took approximately twice as long. The total cost including the Mechanical Turk commission amounted to US $374.

### Preliminary Preferences and Feedback

The main goal of this study was to explore the feasibility of using crowdsourcing to obtain feedback on information presentation options. Thus, we mention the actual results regarding respondents’ preferences primarily for the sake of completeness. Both the Spanish and English survey results indicate that participants largely preferred the format that included bulleted text with an image related to the text. The remaining preferences were evenly distributed among the other three formats (Table 3).

**Table 3:** Comparison of format preferences Spanish-speaking and English-speaking survey participants (total n = 399).

Format	Spanish-speaking (n = 163)		English-speaking (n = 236)	
	n	%	n	%
Bullet + image	106	65.0%	136	57.6%
Bullet alone	22	14%	46	20%
Paragraph with images	27	17%	33	14%
Paragraph alone	7	4%	21	9%

In addition to asking participants to answer the survey, we gave them the opportunity to comment on the survey, which was extremely useful. Several participants provided relevant feedback about the HITs and the value of the health promotion documents. In general, participants seemed to enjoy the task and mentioned that they found it educational or that they thought it was a useful research study. As an example, one participant commented:

My kids’ adult teeth got ruined from giving them bottles at bedtime and it pooling in their mouth. Wish I would have known this back when my kids were little.

Other participants mentioned that they disliked photorealistic images of dental diseases but that they valued the message (translated from Spanish):

The image is too graphic but it is adequate to convey the message.

Although the picture is disgusting, it is real, and it is important so moms can be aware of the consequences of sugar in children’s beverages.

Others commented on specific issues of the Spanish wording, preferring expressions from some dialects of Spanish (eg, Mexican) over others.

## Discussion

Crowdsourcing is a new method for gathering data when human participation is required. Our results show that this technology can be used for gathering useful feedback on the design of health information materials from a large number of participants in a rapid and inexpensive manner, which is in line with results from previous studies in related fields [17-21]. This is particularly important for public health purposes, where materials might not be tested because of time and resource constraints, making it difficult to access a sufficiently large subset of the intended target audience. For example, public health agencies in the United States are bound by federal requirements to provide certain health information to non-English-speaking populations [30], which makes having access to such individuals an important step in adapting health promotion materials. Through crowdsourcing we were able to rapidly recruit a large number of native Spanish speakers, residing in the United States, originating from a large number of countries. In addition to collecting data about preferred formats, we also received feedback regarding the survey itself and useful suggestions from Spanish speakers regarding alternative vocabulary and culturally appropriate images.

An unanticipated side effect of this study was the communication of dental health promotion messages to participants who stated they had previously been unaware of some of the dental health recommendations. Several participants noted their appreciation for participating in an educational HIT. It is conceivable that crowdsourcing can be used not only to test and develop health messages but also to distribute messages to targeted populations. In the ideal case, both purposes could be combined in one crowdsourcing application.

The preliminary results of our survey suggested that both English-speaking and Spanish-speaking participants in the United States preferred a format with bullets and images. The preference for images is consistent with results from prior studies using more traditional methods [4,31,32]. Despite this overall trend, preferences were not unanimous: 17.7% of Spanish speakers and 28.4% of English speakers preferred formats without an image. This variation suggests that in the future, feedback regarding individual messaging formats preferences could be used to inform message tailoring. In addition, some Spanish-speaking participants provided feedback on the wording of the survey, which suggests that crowdsourcing could be used to obtain user feedback to edit terms or phrasing in future versions of the survey.

### Limitations of Crowdsourcing

In line with previous studies, the demographic information collected in this study indicates that the population we reached was younger and more educated than the general population. Reaching primarily younger participants could be helpful when targeting messages pertinent to young parents or young adults in general (eg, on sexually transmitted diseases, drug and alcohol abuse, and injury prevention). Interestingly, Spanish speakers overall reported higher levels of education than English speakers. Clearly, one limitation of the Mechanical Turk recruitment method is the difficulty in reaching populations with low literacy, low computer skills, low educational level, or the elderly, although those are often the populations most in need of health information and support. However, 6%–7% of both the Spanish-speaking and English-speaking participants together had less than high school education. It is possible that, with a sufficiently large sample, valid information about some of the less well-represented demographic groups could be obtained at costs that are still lower than those of traditional surveying methods. Alternative methods to Internet surveying may include crowdsourcing through cell phone text messaging, which may later prove to be an effective way to gain feedback from other hard-to-reach groups.

Given the monetary compensation of Mechanical Turk, it is possible that individuals might try to game the system by answering questions quickly without serious consideration. To guard against this, we required participants to accurately answer questions about the content before the HIT would be accepted. Although there is no guarantee that participants gave true answers, all participants answered the content questions accurately, and their comments suggested that the participants considered their answers carefully.

### Limitations of this Study

The main limitation of this study was that, because it was a pilot study, we did not solicit a statistically significant number of participants to draw conclusions about the responses regarding formatting preferences. Our goal in this study was to investigate how easy or difficult it would be to gain access to specific populations through crowdsourcing. An expanded study is needed to identify whether there are significant differences in messaging preferences between English-speaking and Spanish-speaking populations. Our results suggest that by using Mechanical Turk we will be able to recruit a large sample of participants in a relatively short time and at low costs. In addition, we did not compare the results with more conventional survey methods using convenience samples. However, Mechanical Turk has been compared with traditional experimental paradigms in several previous studies (see above) and has been validated as a way to gather survey responses; moreover, we envision crowdsourcing as a different way to access participants that should not necessarily be compared with conventional survey methods. Our results suggest that the demographics of the surveyed population using crowdsourcing are likely to be *different *from those of convenience samples accessed through more traditional methods. As a result, crowdsourcing provides access to a population not readily available through traditional methods. As a consequence, demographic questions need to be included in the survey and taken into consideration when analyzing the results.

### Conclusions

We used crowdsourcing to recruit a substantial number of English-speaking and Spanish-speaking participants for a survey on health promotion materials in 2 to 4 days for low costs. Results suggest that crowdsourcing could become a valuable research tool in public health communications research.

## Multimedia Appendix 1

Sample task performed by study participant.

## Abbreviations

HIT: human intelligence task
