# Supplementary material for: Using Crowdsourcing Technology for Testing Multilingual Public Health Promotion Materials
Source: J Med Internet Res. 2012 Jun 4;14(3):e79. doi: 10.2196/jmir.2063 (PMC3414863; doi:10.2196/jmir.2063)
Supplement: Supplementary file 1 [file jmir_v14i3e79_app1.pdf]

Crowdsourcing Health Promotion Materials – English Survey

I. Survey Instructions

For this study we will show you a dental health information document in four different formats. Read the documents and then answer the questions.

II. Demographic Questions

1. Is English your native language?
Yes/no

2. What is your gender?
Male/Female

3. What is your age?
18-29 years old
30-40 years old
41-50 years old
51-60 years old
61-70 years old
Older than 70

4. What is your last completed grade in school?
Elementary
Middle School
High School
College
Graduate

5. Do you have a dentist you visit regularly (at least once a year)?
Yes/no

6. When did you last see your dentist?
a) In the past 6months
b) Between 6 months and 1 year
c) In the past 1-2 years
d) More than 2 years ago
e) I've never been to a dentist

III. Sample paragraph with four presented formats, each one followed by topic question. *(The order in which the documents appeared was randomly assigned)*

FORMAT A

Choose beverages for your children that hydrate and contribute to good nutrition. Drinking 8-12 cups of water a day is important and consuming optimally fluoridated water can help prevent tooth decay. A resource list of nutritious foods and beverages that could be sold in schools was developed by the California Center for Public Health Advocacy and may be found by clicking here. The California Center for Public Health Advocacy resource list recommends beverages that may be sold in schools including, beverages that contain at least 50% fruit juice with no added sweeteners, water, low fat milk and non-fat milk. This resource list was used to develop part of the 2001 California law, which establishes nutritional requirements for foods and beverages sold and served to children during breaks and through vending machines.

1. What is this paragraph about?

FORMAT B

- Choose beverages for your children that hydrate and contribute to good nutrition.
- Drinking 8-12 cups of water a day is important and consuming optimally fluoridated water can help prevent tooth decay.
- A resource list of nutritious foods and beverages that could be sold in schools was developed by the California Center for Public Health Advocacy.
- The California Center for Public Health Advocacy resource list recommends beverages that may be sold in schools including, beverages that contain at least 50% fruit juice with no added sweeteners, water, low fat milk and non-fat milk.
- This resource list was used to develop part of the 2001 California law, which establishes nutritional requirements for foods and beverages sold and served to children during breaks and through vending machines.

2. According to this document, how many cups of water should children drink in a day?

FORMAT C

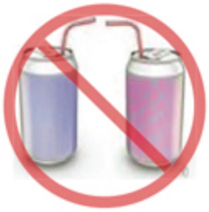

Choose beverages for your children that hydrate and contribute to good nutrition. Drinking 8-12 cups of water a day is important and consuming optimally fluoridated water can help prevent tooth decay. A resource list of nutritious foods and beverages that could be sold in schools was developed by the California Center for Public Health Advocacy and may be found by clicking here. The California Center for Public Health Advocacy resource list recommends beverages that may be sold in schools including, beverages that contain at least 50% fruit juice with no added sweeteners, water, low fat milk and non-fat milk. This resource list was used to develop part of the 2001 California law, which establishes nutritional requirements for foods and beverages sold and served to children during breaks and through vending machines.

3. What kind of beverages does the California Center for Public Health Advocacy recommend?

FORMAT D

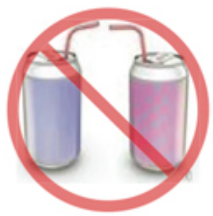

- Choose beverages for your children that hydrate and contribute to good nutrition.
- Drinking 8-12 cups of water a day is important and consuming optimally fluoridated water can help prevent tooth decay.
- A resource list of nutritious foods and beverages that could be sold in schools was developed by the California Center for Public Health Advocacy.
- The California Center for Public Health Advocacy resource list recommends beverages that may be sold in schools including, beverages that contain at least 50% fruit juice with no added sweeteners, water, low fat milk and non-fat milk.
- This resource list was used to develop part of the 2001 California law, which establishes nutritional requirements for foods and beverages sold and served to children during breaks and through vending machines.

4. How much fruit juice should beverages recommended by the California Center for Public Health Advocacy contain?

Please tell us which version of the document you prefer.

A
B
C
D
E

Please tell us the reasons why you prefer it.

____

I. Survey Instructions

En este estudio le mostraremos un documento con información de salud dental en cuatro formatos distintos. Lea los documentos y luego responda las preguntas.

II. Demographic Questions

1. ¿Por favor señale su país de origen?
____

2. ¿Cual es su sexo?
Masculino/Femenino

3. Por favor señale su rango de edad:
18-29 años
30-40 años
41-50 años
51-60 años
61-70 años
Más de 70 años

4. ¿Cual fue el último grado completado en la escuela?

Primaria (6o Grado)
Secundaria (9o Grado)
Preparatoria (12o Grado)
Licenciatura
Maestría
Doctorado

5. ¿Tiene un dentista al que visite regularmente? (al menos una vez al año)
Si/No

6. ¿Cuando fue la última vez que visitó al dentista?
a) durante los últimos 6 meses
b) entre 6 meses a un año atrás
c) entre 1 y 2 años atrás
d) más de 2 años atrás
e) nunca he visitado al dentista

III Sample paragraph with four presented formats, each one followed by topic question. *(The order in which the documents appeared was randomly assigned).*

FORMAT A

Elija bebidas que hidraten y contribuyan a una buena nutrición para sus hijos. Es importante que beban 8 a 12 vasos de agua al día, además, el consumo de agua óptimamente fluorada puede ayudar a prevenir las caries dentales. El Centro para la Defensa de la Salud Pública de California ha desarrollado una lista de alimentos nutritivos y bebidas que pueden ser vendidos en las escuelas. Puede acceder a ella haciendo click aquí. El listado del Centro para la Defensa de la Salud Pública de California recomienda las bebidas que puedan ser vendidas en las escuelas, incluyendo bebidas con al menos un 50% jugo de fruta sin endulzantes añadidos, agua, leche baja en grasa y leche descremada. Esta lista se utilizó para desarrollar parte de la ley 2001 de California, que establece los requerimientos nutricionales para los alimentos y bebidas que se venden y sirven a los niños durante los recreos y por en máquinas expendedoras.

1. ¿De qué se trata este párrafo?

FORMAT B

- Elija bebidas que hidraten y contribuyan a una buena nutrición para sus hijos.
- Es importante que beban 8 a 12 vasos de agua al día, además, el consumo de agua óptimamente fluorada puede ayudar a prevenir las caries dentales.
- El Centro para la Defensa de la Salud Pública de California ha desarrollado una lista de alimentos nutritivos y bebidas que pueden ser vendidos en las escuelas. Puede acceder a ella haciendo click aquí
- El listado del Centro para la Defensa de la Salud Pública de California recomienda las bebidas que puedan ser vendidas en las escuelas, incluyendo bebidas con al menos un 50% jugo de fruta sin endulzantes añadidos, agua, leche baja en grasa y leche descremada.
- Esta lista se utilizó para desarrollar parte de la ley 2001 de California, que establece los requerimientos nutricionales para los alimentos y bebidas que se venden y sirven a los niños durante los recreos y por en máquinas expendedoras.

2. De acuerdo a este documento, ¿Cuántas tazas de agua deben beber al día los niños?

FORMAT C

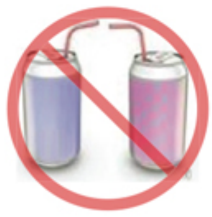

Elija bebidas que hidraten y contribuyan a una buena nutrición para sus hijos. Es importante que beban 8 a 12 vasos de agua al día, además, el consumo de agua óptimamente fluorada puede ayudar a prevenir las caries dentales. El Centro para la Defensa de la Salud Pública de California ha desarrollado una lista de alimentos nutritivos y bebidas que pueden ser vendidos en las escuelas. Puede acceder a ella haciendo click aquí. El listado del Centro para la Defensa de la Salud Pública de California recomienda las bebidas que puedan ser vendidas en las escuelas, incluyendo bebidas con al menos un 50% jugo de fruta sin endulzantes añadidos, agua, leche baja en grasa y leche descremada. Esta lista se utilizó para desarrollar parte de la ley 2001 de California, que establece los requerimientos nutricionales para los alimentos y bebidas que se venden y sirven a los niños durante los recreos y por en máquinas expendedoras.

3. ¿Qué tipo de bebidas recomienda el Centro de California para la Defensa de la Salud Pública?

FORMAT D

- Elija bebidas que hidraten y contribuyan a una buena nutrición para sus hijos.
- Es importante que beban 8 a 12 vasos de agua al día, además, el consumo de agua óptimamente fluorada puede ayudar a prevenir las caries dentales.

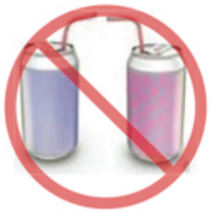

- El Centro para la Defensa de la Salud Pública de California ha desarrollado una lista de alimentos nutritivos y bebidas que pueden ser vendidos en las escuelas. Puede acceder a ella haciendo click aquí
- El listado del Centro para la Defensa de la Salud Pública de California recomienda las bebidas que puedan ser vendidas en las escuelas, incluyendo bebidas con al menos un 50% jugo de fruta sin endulzantes añadidos, agua, leche baja en grasa y leche descremada.
- Esta lista se utilizó para desarrollar parte de la ley 2001 de California, que establece los requerimientos nutricionales para los alimentos y bebidas que se venden y sirven a los niños durante los recreos y por en máquinas expendedoras.

4. ¿Cuanto jugo de frutas deben tener las bebidas recomendadas por el Centro de California para la Defensa de la Salud Pública?

IV. Questions about document preferences

¿Qué versión del documento prefiere?

A
B
C
D

Por favor señale las razones de por qué lo prefiere.

____
